# Dynamic Consistency-Aware Multi-View Learning with SAM3 for 3D Medical Image Segmentation

**DOI:** 10.3390/s26185753

**Published:** 2026-09-10

**Authors:** Xuan Chen, Shaolong Chen

**Affiliations:** 1 School of Computer Sciences, Universiti Sains Malaysia, George Town 11600, Malaysia; chenxuan9001@163.com; 2 School of Electronics and Communication Engineering, Sun Yat-sen University, Guangzhou 510006, China

**Keywords:** 3D medical image segmentation, dynamic consistency-aware, multi-view learning, SAM3, edge detail preservation

## Abstract

Three-dimensional medical image segmentation is critical for clinical diagnosis and treatment planning. Medical images typically exhibit heterogeneous characteristics of low inter-slice resolution and high in-plane resolution, leading 3D CNNs to lose fine edge details via cross-slice feature averaging and traditional 2D slice-based methods to produce discontinuous results due to neglected inter-slice spatial relationships. Multi-view learning shows potential in addressing these issues but lacks sample-adaptive dynamic consistency awareness, as conventional fixed-weight fusion cannot adaptively calibrate view discrepancies for heterogeneous medical data. To tackle this, we propose a dynamic consistency-aware multi-view framework enhanced with SAM3 for 3D medical image segmentation. The framework embeds pairwise cross-view similarity-driven dynamic gating into the multi-view pipeline: decomposing 3D images into three orthogonal views, extracting fine-grained features with a frozen SAM3 encoder, and using a dynamic consistency-aware fusion module that computes pairwise view similarity to dynamically calibrate resolution-induced discrepancies, capture inter-slice and cross-view spatial dependencies, and reconstruct unified 3D features, which are then mapped to segmentation masks via a decoder. Experiments on Automated Cardiac Diagnosis Challenge (ACDC) and infrapatellar fat pad (IPFP) datasets demonstrate that our method achieves superior segmentation accuracy over state-of-the-art approaches, with promising technical performance that supports further multicenter prospective clinical validation.

## 1. Introduction

Medical image segmentation plays an indispensable role in modern clinical workflows, such as disease diagnosis, treatment planning, and prognosis evaluation. By segmenting target anatomical structures from medical images, clinicians can obtain quantitative information to support clinical decision-making [1,2]. Among various medical imaging modalities, 3D volumetric data can provide comprehensive spatial information on anatomical structures, which is crucial for accurate clinical assessment [3,4]. However, 3D medical image segmentation is a challenging task due to the high dimensionality of data, complex anatomical structures, varying intensity distributions, and the presence of noise and artifacts [5,6].

In recent years, deep learning-based methods have achieved remarkable success in medical image segmentation [7,8]. However, these methods are severely challenged by the inherent heterogeneous characteristics of medical images: low inter-slice resolution and high in-plane resolution. Traditional 3D convolutional neural networks (CNNs), such as VNet [9], 3D U-Net [10], and 3D TransUNet [11], directly process 3D volumetric data but tend to average cross-slice features due to the resolution heterogeneity, leading to severe loss of fine edge details of tumors and organs critical for clinical diagnosis. On the other hand, 2D CNN-based methods [12,13] ignore inter-slice spatial relationships, resulting in discontinuous segmentation results. As a powerful paradigm for feature representation enhancement, multi-view learning has been widely explored in medical image segmentation by leveraging complementary information from multiple orthogonal views [14,15,16]. However, existing multi-view learning methods for medical image segmentation suffer from a core limitation: lack of dynamic consistency awareness. They simply fuse slice-level features across views without adaptively calibrating view-specific discrepancies caused by medical image resolution heterogeneity and fail to dynamically capture inter-view/inter-slice spatial consistency, leading to suboptimal feature representation and discontinuous segmentation results. Notably, the emerging segment anything model 3 (SAM3) excels in extracting fine-grained 2D features [17], which is promising for addressing edge detail loss, but lacks effective integration with dynamic consistency-aware multi-view learning to solve the above limitations. To tackle these critical issues—edge detail loss of 3D CNNs, segmentation discontinuity of single-view 2D methods, and insufficient dynamic consistency awareness of traditional multi-view learning—we propose a dynamic consistency-aware multi-view learning framework enhanced with SAM3. This framework embeds dynamic consistency awareness into the entire multi-view learning pipeline, integrating SAM3’s fine-grained feature extraction capability to ensure 3D representation consistency and edge detail preservation.

The Segment Anything Model (SAM) is a powerful foundation model for image segmentation [18,19], showing excellent performance in extracting fine-grained 2D features; this advantage is particularly valuable for complementing dynamic consistency-aware multi-view learning in addressing edge detail loss caused by medical image heterogeneity. SAM3, the latest version of SAM, further enhances feature representation capability and efficiency. Multi-view learning, as a core paradigm, aims to aggregate complementary information from multiple views to improve model performance, but its effectiveness in medical image segmentation is constrained by the lack of dynamic consistency awareness. Inspired by SAM3’s fine-grained feature extraction strength and the potential of dynamic consistency-aware multi-view learning, we propose to embed dynamic consistency awareness into the entire multi-view learning process: using SAM3 to extract high-quality features for each view and designing a dynamic consistency-aware fusion module to adaptively optimize cross-view feature interaction. This integration enables the framework to fully exploit multi-view complementary information while dynamically maintaining feature consistency and preserving edge details, thereby solving the core challenges of existing 3D medical image segmentation methods. Rigorous robustness and generalization evaluation is a necessary prerequisite for clinical translation of medical foundation models [20,21], and our work follows this principle to design and evaluate the proposed framework.

Our main contributions are summarized as follows:

(1) We propose a dynamic consistency-aware multi-view framework for heterogeneous medical images, which incorporates pairwise cross-view similarity-driven adaptive fusion throughout the pipeline, overcoming the fixed-weight fusion limitation of traditional multi-view learning methods.
(2) We integrate SAM3 into the framework, utilizing its fine-grained 2D feature extraction to acquire high-quality view-specific features, facilitating dynamic consistency maintenance and mitigating edge detail loss in heterogeneous data.
(3) We design a dedicated dynamic consistency-aware fusion module that adaptively calibrates resolution-induced view discrepancies, captures inter-slice and cross-view spatial dependencies, and reconstructs consistent 3D features to resolve segmentation discontinuity.
(4) Extensive experiments on public datasets confirm that our method outperforms state-of-the-art approaches in accuracy, verifying the effectiveness of dynamic consistency-aware multi-view learning and its clinical translational potential.

## 2. Methods

**Figure 1:**
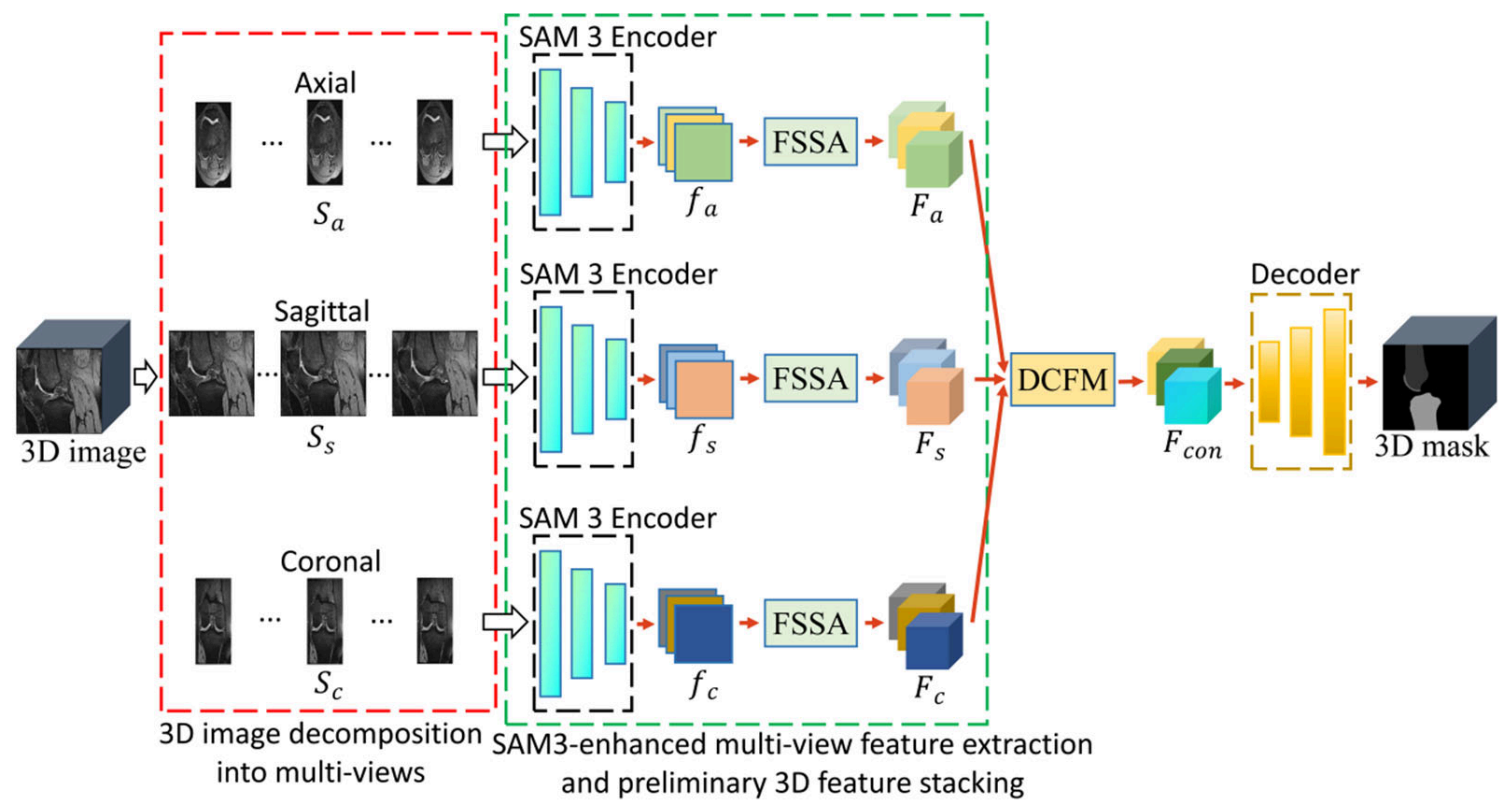
The overall architecture of the proposed dynamic consistency-aware multi-view framework enhanced with SAM3. FSSA: Preliminary 3D feature stacking and spatial alignment. DCFM: Dynamic consistency-aware fusion for multi-view learning.

The overall architecture of the proposed dynamic consistency-aware multi-view framework enhanced with SAM3 is illustrated in Figure 1. It embeds dynamic consistency awareness into the entire multi-view learning pipeline and enhances feature quality with SAM3. The framework consists of four key components, as detailed below:

(1) 3D image decomposition into multiple views: serving as the view initialization for dynamic consistency-aware learning, this step ensures complementary spatial information conducive to subsequent consistency maintenance.
(2) SAM3-enhanced multi-view feature extraction and preliminary 3D feature stacking: extracting high-quality fine-grained view-specific features and forming preliminary 3D feature volumes.
(3) Dynamic consistency-aware fusion for multi-view learning: adaptively calibrating view discrepancies and capturing spatial dependencies to reconstruct consistent 3D features.
(4) Decoder: mapping consistent 3D features to final segmentation masks while preserving edge details and spatial continuity.

Each component is elaborated in detail as follows.

### 2.1. 3D Image Decomposition into Multi-Views

Given a 3D medical image volume $V\in R^{H\times W\times D}$, where H, W and D represent the height, width, and depth of the volume, respectively. To address resolution heterogeneity related to direction, we first use third-order B-spline interpolation to resample all volumes to isotropic voxel spacing and apply Gaussian smoothing after resampling to suppress interpolation artifacts. Then, we decompose the resampled volume into three orthogonal views (sagittal, coronal, and axial) to capture complementary spatial information from different angles. Specifically,

Sagittal view: A set of 2D slices $S_{s}\;=\;s_{s,\;1},\;s_{s,\;2},\;\ldots ,\;s_{s,\;H}$ where each slice $s_{s,\;i}\;\in \;R^{W\;\times \;D}$ is obtained by fixing the height dimension.

Coronal view: A set of 2D slices $S_{c}\;=\;s_{c,\;1},\;s_{c,\;2},\;\ldots ,\;s_{c,\;W}$ where each slice $s_{c,\;j}\;\in \;R^{H\;\times \;D}$ is obtained by fixing the width dimension.

Axial view: A set of 2D slices $S_{a}\;=\;s_{a,\;1},\;s_{a,\;2},\;\ldots ,\;s_{a,\;D}$ where each slice $s_{a,\;k}\;\in \;R^{H\;\times \;W}$ is obtained by fixing the depth dimension.

### 2.2. SAM3-Enhanced Multi-View Feature Extraction and Preliminary 3D Feature Stacking

View feature extraction is a core prerequisite for dynamic consistency-aware multi-view learning, and feature quality directly determines the effectiveness of subsequent consistency calibration. This component focuses on extracting fine-grained view-specific features via SAM3 and constructing preliminary 3D feature volumes with consistent spatial dimensions.

#### 2.2.1. Fine-Grained Feature Extraction with SAM3

In our framework, only the SAM3 image encoder is adopted to extract general fine-grained features from 2D medical slices. This is because the image encoder is optimized for capturing detailed object boundaries, which can compensate for edge detail loss in heterogeneous medical data and provide cross-view consistent feature descriptors. We do not rely on specific prompts to ensure the framework’s generalization ability.

Let $F_{\mathrm{SAM}3}()$ denote the feature extraction function of the SAM3 image encoder. For each slice $s_{s,\;i}$ in the sagittal view, the extracted 2D feature map is $f_{s,\;i}=F_{\mathrm{SAM}3}(s_{s,\;i})\;\in \;R^{h_{s}\;\times \;w_{s}\;\times \;c}$, where $h_{s}$, $w_{s}$, and c are the height, width, and number of channels of the feature map, respectively. Similarly, for each slice in the coronal and axial views, the extracted 2D features are $f_{c,\;i}$ and $f_{a,\;i}$, respectively.

#### 2.2.2. Preliminary 3D Feature Stacking and Spatial Alignment

To construct 3D feature representations for each view, we stack the 2D feature maps of each view along their respective dimensions:

Sagittal view: Stack along the height dimension to form $F_{s}\;\in \;R^{H\;\times \;h_{s}\;\times \;w_{s}\;\times \;c}$.

Coronal view: Stack along the width dimension to form $F_{c}\;\in \;R^{W\;\times \;h_{c}\;\times \;w_{c}\;\times \;c}$.

Axial view: Stack along the depth dimension to form $F_{a}\;\in \;R^{D\;\times \;h_{a}\;\times \;w_{a}\;\times \;c}$.

Due to differences in the original stacking dimensions, the three feature volumes have inconsistent spatial sizes. To enable cross-view feature interaction and consistency calibration, we perform transformation and interpolation on $F_{c}$ and $F_{a}$ to unify their spatial sizes with that of $F_{s}$.

Feature stacking is only the first step of 3D volume construction. After stacking, subsequent 3D convolutions in the projection layer, dynamic cross-view attention in DCFM, and 3D decoding operations all explicitly model local inter-slice spatial dependencies via 3D convolution; the SAM3 encoder itself does not model inter-slice relationships, with inter-slice continuity guaranteed by these follow-up 3D computation modules.

### 2.3. Dynamic Consistency-Aware Fusion for Multi-View Learning

The preliminary 3D feature volumes $F_{s}$, $F_{c}$, and $F_{a}$ capture complementary spatial information and fine-grained edges, but still suffer from view-specific discrepancies and incomplete cross-view/inter-slice spatial dependencies. To address these issues, we design a dedicated dynamic consistency-aware fusion module, which adaptively calibrates discrepancies and captures spatial dependencies to reconstruct a unified, consistent 3D feature representation. Following the standard definition of dynamic multi-view fusion, the “dynamic” property of our module refers to fusion weights being adaptively generated based on the cross-view feature similarity of each individual input, rather than using fixed global parameters identical for all samples. This dynamic behavior is naturally implemented via pairwise cross-view spatial attention. The module consists of two key steps: dynamic cross-view attention calibration and channel-wise consistency enhancement.

#### 2.3.1. Dynamic Cross-View Attention Calibration

This step adaptively captures spatial interdependencies between different views and calibrates view-specific discrepancies induced by resolution heterogeneity using spatial attention. For each pair of feature volumes, we flatten the spatial dimensions into a single sequence dimension $N\;=\;H\;\times \;h_{s}\;\times \;w_{s}$, then compute cross-view spatial attention to aggregate complementary information and align inconsistent features. For example, the attention calibration between $F_{s}$ and $F_{c}$ is calculated as:

(1) $$A_{s,\;c}\;=\;\mathrm{Softmax}(\frac{F_{s}F_{c}^{T}}{\sqrt{c}})$$

(2) $$F_{s\leftarrow c}=A_{s,\;c}F_{c}$$

where Softmax is the softmax activation function. $A_{s,c}\in R^{N\times N}$ is the spatial attention matrix reflecting voxel-wise similarity between $F_{s}$ and $F_{c}$, and $F_{s\leftarrow c}$ denotes the features of $F_{c}$ aggregated into $F_{s}$. We repeat this operation for all view pairs ($F_{s}\text{↔}F_{a}$, $F_{c}\text{↔}F_{a}$) to obtain aggregated features $F_{s\leftarrow a}$, $F_{c\leftarrow s}$, $F_{c\leftarrow a}$, $F_{a\leftarrow s}$, $F_{a\leftarrow c}$.

Subsequently, we fuse the original features and aggregated cross-view features via concatenation to retain both view-specific information and cross-view complementary information:

(3) $$\;F_{s}^{\prime }=\;\mathrm{Concat}(F_{s},\;F_{s\leftarrow c},\;F_{s\leftarrow a})$$

(4) $$F_{c}^{\prime }=\mathrm{Concat}(F_{c},\;F_{c\leftarrow s},\;F_{c\leftarrow a})$$

(5) $$F_{a}^{\prime }=\mathrm{Concat}(F_{a},\;F_{a\leftarrow s},\;F_{a\leftarrow c})$$

where $F_{s}^{\prime }$, $F_{c}^{\prime }$ and $F_{a}^{\prime }$ denote the feature maps of $F_{s}$, $F_{c}$ and $F_{a}$ after dynamic cross-view attention calibration, respectively. Concat represents the channel concatenation operation.

In our implementation, the SAM3 encoder produces feature maps of size 24 × 24 for each 336 × 336 slice. With *H* = 32, the *N* = *H* × 24 × 24 = 18,432. The attention matrix is *N* × *N* (≈3.40 × 10^8^ elements), requiring 680 MB in FP16. The three view pairs are processed sequentially to avoid peak memory overflow, and on an NVIDIA RTX 4090, the additional computation takes less than 1 s per iteration, which is acceptable for offline training and inference.

#### 2.3.2. Channel-Wise Consistency Enhancement

To ensure that the fused features maintain consistent responses across views for the same anatomical structure, we apply a channel attention module to each fused feature volume. This module adaptively adjusts channel weights, highlighting critical feature channels and suppressing redundant ones.

The channel attention mask is computed as follows:

(6) $$M_{s}\;=\;\mathrm{Sigmoid}({W_{s,\;2}\cdot \mathrm{ReLU}(W}_{s,\;1}\cdot \mathrm{GAP}(\;F_{s}^{\prime })\;+\;b_{s,\;1})\;+\;b_{s,\;2})$$

(7) $$M_{c}\;=\mathrm{Sigmoid}({W_{c,\;2}\cdot \mathrm{ReLU}(W}_{c,\;1}\cdot \mathrm{GAP}(\;F_{c}^{\prime })+b_{c,\;1})+b_{c,\;2})\;$$

(8) $$M_{a}=\mathrm{Sigmoid}({W_{a,\;2}\cdot \mathrm{ReLU}(W}_{a,\;1}\cdot \mathrm{GAP}(\;F_{a}^{\prime })+b_{a,\;1})+b_{a,\;2})\;$$

where GAP() denotes global average pooling. $W_{s,\;1}$, $W_{s,\;2}$, $W_{c,\;1}$, $W_{c,\;2}$, $W_{a,\;1}$ and $W_{a,\;2}$ are learnable weight matrices. $b_{s,\;1}$, $b_{s,\;2}$, $b_{c,\;1}$, $b_{c,\;2}$, $b_{a,\;1}$ and $b_{a,\;2}$ are bias terms. The calibrated feature volume is obtained by element-wise multiplication of the fused feature and the attention mask:

(9) $$F_{s,\;\mathrm{att}}\;=\;M_{s}\cdot F_{s}^{\prime }$$

(10) $$F_{c,\;\mathrm{att}}={\;M}_{c}\cdot F_{c}^{\prime }$$

(11) $$F_{a,\;\mathrm{att}}={\;M}_{a}\cdot F_{a}^{\prime }$$

After channel-wise consistency enhancement, we concatenate the three calibrated feature volumes $\left(F_{s,\;\mathrm{att}},\;F_{c,\;\mathrm{att}},\;F_{a,\;\mathrm{att}}\right)$ to obtain the final consistent 3D feature volume $F_{\mathrm{con}}\;\in {\;R}^{H\;\times \;h_{s}\;\times \;w_{s}\;\times \;9c}$.

### 2.4. Decoder

The decoder is designed to map the consistent feature volume $F_{\mathrm{con}}$(size $H\;\times \;h_{s}\;\times \;w_{s}\;\times \;9c$) to the final fine-grained segmentation mask, while preserving the edge details extracted by SAM3 and the spatial consistency reconstructed by the fusion module.

To obtain hierarchical multi-scale representations, we apply four parallel 1 × 1 × 1 convolutions to compress $F_{\mathrm{con}}$ into four lightweight volumes, each of size $H\;\times \;h_{s}\;\times \;w_{s}\;\times \;c^{\prime }$. Since the *H*-dimension remains uncompressed, we employ bilinear interpolation to resize the ($h_{s}$, $w_{s}$) spatial dimensions of these four volumes to the original (*W*, *D*) space, yielding scales of (*H*, *W*, *D*), (*H*, *W*/2, *D*/2), (*H*, *W*/4, *D*/4), and (*H*, *W*/8, *D*/8).

The feature resized to (*H*, *W*/8, *D*/8) is used as the initial input to the decoder backbone, while the other three serve as lateral connections. The decoder core consists of stacked transposed convolutions. At each upsampling stage, we concatenate the upscaled feature with the corresponding lateral feature map of the same spatial size.

Finally, a 1 × 1 × 1 convolution reduces the channel dimension to the number of target classes, followed by a softmax activation to produce voxel-wise probability maps. The final segmentation mask is obtained via argmax over the class channel.

## 3. Experiments and Results

### 3.1. Experimental Datasets

To verify the effectiveness and generalization performance of our method, extensive experiments were carried out on two 3D medical image segmentation datasets: the Automated Cardiac Diagnosis Challenge (ACDC) [22] and the Infrapatellar Fat Pad (IPFP) dataset. For the ACDC dataset, we used 100 cases with physician-annotated ground truths (right ventricle (RV), myocardium (Myo), and left ventricle (LV)). We used 70 cases as the training set, 10 cases as the validation set, and 20 cases as the test set. Subsequently, all volumes were uniformly cropped or padded to a size of 32 × 336 × 336. For the IPFP dataset, the case images were derived from the OAI public database (https://nda.nih.gov/oai/, accessed on 6 May 2022). Annotations for the IPFP dataset were performed by two radiologists with five years of imaging experience. All annotation discrepancies were resolved via consensus review by a senior radiologist with 15 years of experience. We used 138 cases for training, 25 for validation, and 57 for testing. All volumes were uniformly cropped or padded to a size of 32 × 336 × 336. The following data augmentation methods were adopted: slight translation, scaling, rotation, and flipping. Prior to training and testing, the data samples were normalized to zero mean and unit variance.

### 3.2. Implementation Details

The experiment was run on two NVIDIA RTX 4090 GPUs. The SAM3 encoder used pre-trained weights (sam3.pt). All methods were trained with the Adam optimizer, a batch size of 2, a learning rate of 5 × 10^−4^, and a maximum of 1000 epochs. Early stopping was set to 10. During training, we froze the parameters of the SAM3 encoder. The loss function used in each method was a combination of binary cross-entropy (weight 0.5) and Dice loss (weight 1).

All methods used exactly the same training/validation/test splits and the same preprocessing as our proposed method, ensuring a fully fair comparison. The SAM3 baseline used exactly the same frozen sam3.pt encoder checkpoint as our method and did not use any box/point prompt input for fully automatic segmentation.

### 3.3. Evaluation Metrics

In line with the common evaluation standards in the field of medical image segmentation, this paper used the Dice coefficient (Dice) and the 95th percentile Hausdorff distance (HD95) to evaluate the performance of the segmentation method.

### 3.4. Ablation Experiment Results

To verify the effectiveness of each component, we conducted ablation studies on the IPFP dataset. The ablation experiments focused on validating the impact of key components on addressing resolution heterogeneity, preserving edge details, and ensuring segmentation continuity. The ablation experiments included five variants of the proposed method:

Variant 1: Without multi-view learning (using only single axial view features, abandoning multi-view complementary information).

Variant 2: Multi-view learning without dynamic consistency awareness (simple concatenation of three view features, representing traditional multi-view fusion).

Variant 3: Using a traditional CNN (ResNet50) instead of SAM3 for multi-view feature extraction (multi-view learning with low-quality view features).

Variant 4: Without dynamic cross-view attention calibration in the consistency modeling module (multi-view learning enhanced with incomplete dynamic consistency).

Variant 5: Without channel-wise consistency enhancement in the consistency modeling module (multi-view learning enhanced with incomplete dynamic consistency).

The ablation results are shown in Table 1. It can be seen that the proposed method achieves the best performance in terms of Dice. Key observations are as follows: (1) Variant 3 (ResNet50 instead of SAM3) exhibits the largest performance drop, confirming that SAM3’s strong fine-grained feature extraction is the critical foundation for high performance. (2) Variant 1 (without multi-view learning) shows a significant performance drop, confirming that multi-view learning provides essential complementary spatial information. (3) Variant 2 performs worse than the full model, demonstrating that dynamic consistency awareness is the key to enhancing multi-view learning’s performance in handling heterogeneous medical data, effectively solving the consistency deficiency in traditional multi-view learning. (4) Variants 4 and 5 show small but consistent performance drops, confirming that both cross-view spatial attention calibration and channel-wise consistency enhancement are effective complementary components of dynamic consistency-aware fusion.

### 3.5. Experimental Results

To verify the superiority of our method, we compared it with current mainstream medical image segmentation methods, including: V-Net [9], 3D U-Net [10], Med-SA [23], 3D MFA [24], and SAM3 [17].

**Figure 2:**
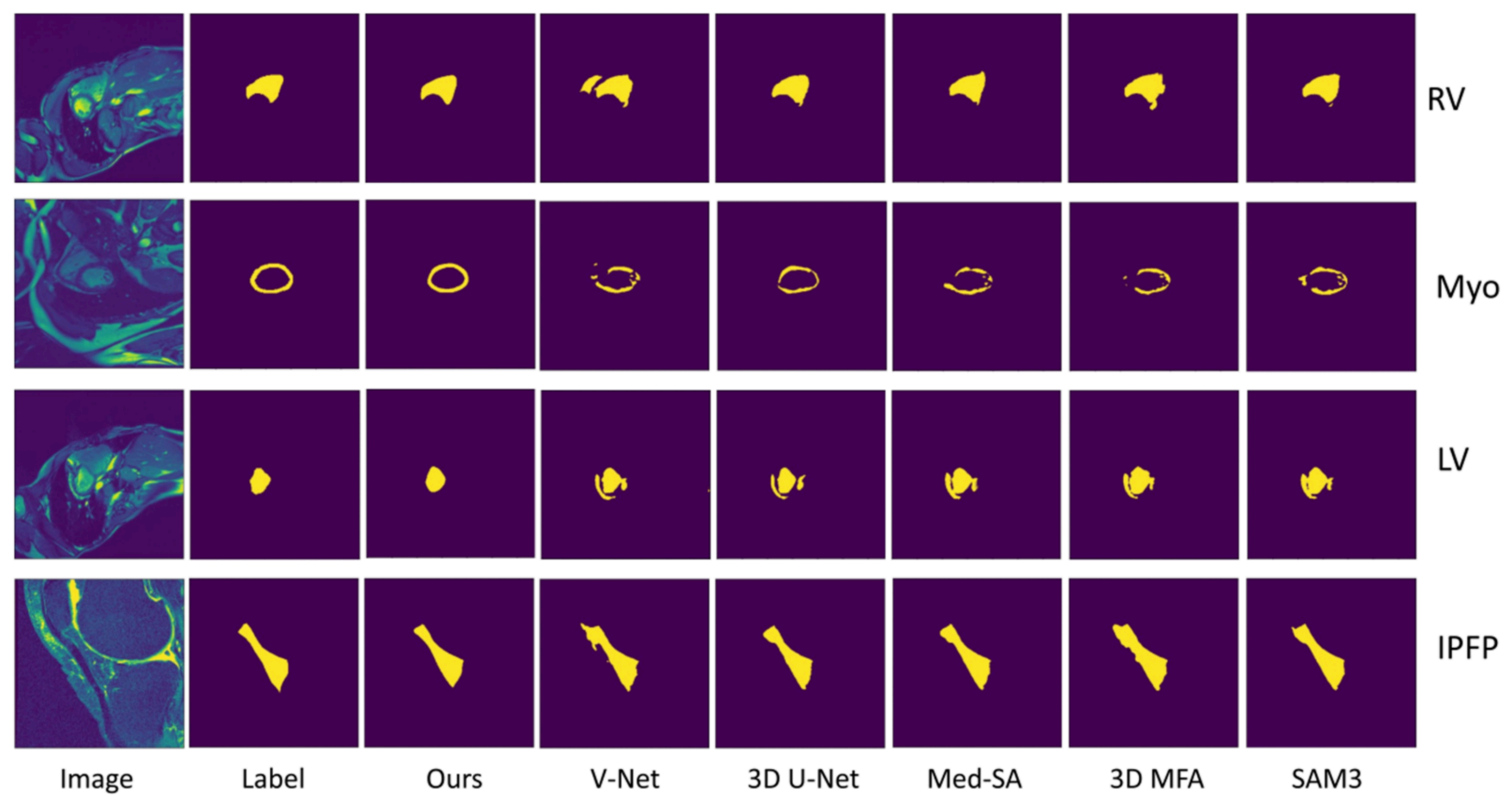
The qualitative comparison results of each method on the ACDC and IPFP datasets.

The quantitative evaluation results of each method on the ACDC and IPFP datasets are shown in Table 2 and Table 3, respectively. It can be seen from the tables that our method achieves the best performance across all evaluation metrics on the two datasets. Figure 2 shows the qualitative comparison results of each method on the ACDC and IPFP datasets. Our method achieves more continuous segmentation and retains finer edge details compared with other methods.

## 4. Conclusions

In this paper, we propose a dynamic consistency-aware multi-view learning framework enhanced with SAM3 for 3D medical image segmentation, aiming to address the core challenges of existing methods: edge detail loss of 3D CNNs, segmentation discontinuity of single-view 2D methods, and the lack of dynamic consistency awareness of traditional multi-view learning. The framework embeds dynamic consistency awareness into the entire multi-view learning pipeline, decomposing 3D medical images into three orthogonal views, extracting SAM3-enhanced fine-grained view features, fusing multi-view features via a dynamic consistency-aware module, and generating fine-grained segmentation masks via a decoder. Extensive experiments on the ACDC and IPFP datasets demonstrate that the proposed method outperforms state-of-the-art approaches in segmentation accuracy. Our method exhibits promising performance for 3D medical image segmentation, which facilitates further multi-center prospective clinical validation.

## Figures and Tables

**Table 1:** The ablation experiment results on the IPFP dataset.

	Variant 1	Variant 2	Variant 3	Variant 4	Variant 5	Ours
Dice	0.903	0.908	0.899	0.914	0.913	0.916

**Table 2:** The quantitative evaluation results of each method on the ACDC dataset.

		V-Net	3D U-Net	Med-SA	3D MFA	SAM3	Ours
RV	Dice	0.901	0.899	0.908	0.910	0.917	0.932
	HD95	6.46	6.38	5.96	5.45	5.11	4.86
Myo	Dice	0.862	0.864	0.871	0.874	0.881	0.889
	HD95	2.96	2.85	2.76	2.70	2.63	2.52
LV	Dice	0.940	0.942	0.951	0.949	0.953	0.961
	HD95	3.46	3.38	3.15	2.91	2.86	2.65

**Table 3:** The quantitative evaluation results of each method on IPFP dataset.

	V-Net	3D U-Net	Med-SA	3D MFA	SAM3	Ours
Dice	0.885	0.882	0.896	0.894	0.906	0.916
HD95	5.43	5.37	5.10	4.97	4.83	4.71

## Data Availability

The data presented in this study are derived from public domain resources. Download the ACDC dataset from https://www.creatis.insa-lyon.fr/Challenge/acdc/ (accessed on 20 May 2022). The images of the IPFP dataset are downloaded from https://nda.nih.gov/oai/ (accessed on 6 May 2022).
